# Adding autogenic drainage to chest physiotherapy after upper abdominal surgery: effect on blood gases and pulmonary complications prevention. Randomized controlled trial

**DOI:** 10.1590/1516-3180.2021.0048.0904221

**Published:** 2021-11-15

**Authors:** Mona Mohamed Taha, Ramy Salama Draz, Mohamed Mostafa Gamal, Zizi Mohamed Ibrahim

**Affiliations:** I MD, PhD. Associate Professor, Department of Rehabilitation, College of Health and Rehabilitation Sciences, Princess Nourah bint Abdulrahman University, Riyadh, Kingdom of Saudi Arabia; and Assistant Professor, Department of Cardiovascular/Respiratory Disorders and Geriatrics, Faculty of Physical Therapy, Cairo University, Cairo, Egypt.; II MD, PhD. Assistant Professor, Department of Cardiovascular/Respiratory Disorders and Geriatrics, Faculty of Physical Therapy, Cairo University, Cairo, Egypt.; III PT, MD. Physiotherapist, Kasr Al-Ainy Teaching Hospital, Giza, Egypt.; IV MD, PhD. Assistant Professor, Department of Surgery, Faculty of Physical Therapy, Cairo University, Cairo, Egypt; and Associate Professor, Department of Rehabilitation, College of Health and Rehabilitation Sciences, Princess Nourah bint Abdulrahman University, Riyadh, Kingdom of Saudi Arabia.

**Keywords:** Blood gas analysis, Postoperative complications, Physical therapy modalities, Autogenic drainage, Blood gases, Pulmonary complications, Physiotherapy techniques, Upper abdominal surgery

## Abstract

**BACKGROUND:**

Hypoxemia and pulmonary complications are common after upper abdominal surgery (UAS).

**OBJECTIVE:**

To examine whether inclusion of autogenic drainage (AD) in chest physiotherapy after UAS confers additional benefits in improving blood gases and reducing postoperative pulmonary complications (PPCs).

**DESIGN AND SETTING:**

Randomized controlled study conducted at Kasr Al-Ainy teaching hospital, Egypt.

**METHODS:**

A randomized controlled trial was conducted on 48 subjects undergoing elective UAS with high risk of developing PPCs. The study group received AD plus routine chest physiotherapy (deep diaphragmatic breathing, localized breathing and splinted coughing) and the control group received routine chest physiotherapy only. The outcomes included arterial blood gases measured at the first and seventh postoperative days, incidence of PPCs within the first seven days and length of hospital stay.

**RESULTS:**

Baseline characteristics were similar between groups. In the AD group, SaO_2_, PaO_2_, PaCO_2_ and HCO_3_ significantly improved (P < 0.05) while in the physiotherapy group, only SaO_2_ and PaO_2_ significantly improved (P < 0.05). Nonetheless, significant differences in post-treatment SaO_2_ and PaO_2_ between the groups were observed. The overall incidence of PPCs was 16.66% (12.5% in the AD group and 20.8% in the physiotherapy group) (absolute risk reduction -8.3%; 95% confidence interval, CI, -13.5 to 29.6%), with no significant difference between the groups. The AD group had a significantly shorter hospital stay (P = 0.0001).

**CONCLUSION:**

Adding AD to routine chest physiotherapy after UAS provided a favorable blood gas outcome and reduced the length of hospital stay. It tended to reduce the incidence of PPCs.

**TRIAL REGISTRATION:**

ClinicalTrials.gov: NCT04446520.

## INTRODUCTION

Surgery and postoperative care have become important in healthcare worldwide, given that nearly 234 million patients undergo operations every year.^1^ In developed countries, the most common major surgical procedure is upper abdominal surgery (UAS).^2^ After UAS, postoperative pulmonary complications (PPCs) contribute to poor patient outcomes, significant lengthening of hospital stay, increased readmissions and elevation of healthcare costs, and may constitute an important and significant cause of morbidity and mortality.^3^ Eighty-five percent of PPCs occur during the first three days after surgery^4^ and are partially caused by earlier pathophysiological reductions of lung volumes postoperatively.^5^ If these conditions persist, they may lead to severe hypoxemia, atelectasis and pneumonia.^6^

The presence of preoperative risk factors like old age, malnutrition, smoking, obesity and clinical lung diseases increases the incidence of PPCs among subjects undergoing UAS. Other anesthetic and surgical factors like the duration and type of surgery also contribute to PPC development.^7^

Lunardi et al.^8^ reported that after UAS, impairment of respiratory capacity occurred. This was correlated with a reduction in chest wall volume of 22% and breathing pattern modification due to a decline in the diaphragmatic contribution by 28% to 40%. Reduced lung volumes after surgery are associated with accumulation of pulmonary secretions, which can favor bacterial colonization and development of postoperative pneumonia.^9^ Moreover, inability to cough and ineffective coughing by patients after surgery have been found to contribute to the basis of the pathophysiology for PPCs, as this can cause excessive accumulation of expectorations and elevate the risk of developing pulmonary infections and obstructive atelectasis. Additionally, the decreases in alveolar ventilation and in clearance of bronchial secretions also reduce the clearance of carbon dioxide (CO_2_), thus causing hypercarbia, acidosis and, usually, moderate hypoxemia.^10^

Since the beginning of the 20^th^ century, chest physiotherapy has commonly been used for prevention and management of PPCs such as atelectasis, retained secretions, bronchopulmonary infection and pneumonia.^3^ Postoperative chest physiotherapy improves lung volume and ventilation-perfusion matching, facilitates mucociliary clearance and reduces pain.^11^

Different alternatives for advanced airway clearance techniques have been developed to improve the efficiency of airway clearance and encourage patient autonomy. These have included autogenic drainage (AD),^12^ which depends more on breathing pattern control by the individual to facilitate airway clearance.^13^ AD consists of controlled tidal breathing that is practiced at different levels of lung volume. In this, the subject self-adjusts the force or velocity of the expiratory airflow at different levels of inspiration in order to reach the maximum possible airflow generated in the bronchi, without resulting in airway collapses during coughing.^12^ AD has the advantage that it is tolerable and can be self-administered and performed from a seated position.^14^

Although there is widespread use of different chest physiotherapy methods after UAS, the impact of each technique on blood gases and prevention of PPCs has not been illustrated. Only limited clinical trials using the autogenic drainage technique after surgical procedures have been presented in the literature and there has not been any assessment of this technique in the population undergoing UAS.

## OBJECTIVE

The aim of this study was to evaluate the effect of adding AD to routine chest physiotherapy, with regard to improving blood gases and decreasing the incidence of PPCs and length of hospital stay in the postoperative period, among subjects undergoing UAS.

## METHODS

### Design and sample

A randomized controlled trial was conducted on 60 obese subjects of both sexes who underwent UAS in Kasr Al-Ainy teaching hospital. This study was approved by the Institutional Review Board of the Faculty of Physical Therapy, Cairo University, under the number P.T.REC/012/001740, on October 1, 2017, and it was conducted in accordance with the principles of the Declaration of Helsinki. A verbal explanation of the trial was provided to eligible subjects and informed consents were obtained.

Subjects with ages from 50 to 60 years and body mass index (BMI) from 30 to 40 who underwent elective UAS were included in the study. In addition, the following procedure-related inclusion criteria were applied: expected duration of surgery ≥ 120 minutes; abdominal incision more than five centimeters above or extending above the navel; planned postoperative admission to the surgical ward; and anticipated length of hospital stay after surgery of more than six days. The exclusion criteria were situations in which subjects underwent any of the following procedures: laparoscopic surgery, lower abdominal surgery, emergency surgery, esophageal surgery or organ transplantation. In addition, subjects who were unable to follow the physiotherapy instructions, received preoperative physiotherapy or persisted with a requirement for invasive mechanical ventilation for more than 24 hours postoperatively were also excluded.

According to these inclusion criteria, the subjects were considered to be at high risk of developing PPCs because they had histories of cigarette smoking, long durations of surgery (> 120 minutes) and obesity (BMI > 27 kg/m^2^).^7^ They were randomly assigned equally to two groups: the study group included 30 subjects who received routine chest physiotherapy (deep diaphragmatic breathing exercises, localized breathing exercises and splinted coughing) plus the AD technique; and the physiotherapy group included 30 subjects who received routine chest physiotherapy only.

Randomization of eligible participants who were present in the waiting list for surgery was done by means of sequentially numbered sealed opaque envelopes that contain allocation cards. These envelopes had been sealed by an independent administrator who did not participate in the trial. From these sealed opaque envelopes, an independent nurse selected the participant for group allocation. The allocation sequence was 1:1, and this was enabled through a web-based computer-generated blocked random number table (https://randomization.com).

### Outcome measurements

The primary outcome measurements were changes to arterial blood gases through the following parameters: arterial oxygen saturation (SaO_2_), partial pressure of oxygen (PaO_2_), partial pressure of carbon dioxide (PaCO_2_), pH and bicarbonate (HCO_3_). These were measured on the first and seventh days postoperatively, at room air temperature.

The secondary outcome measurements included: (A) the incidence of PPCs (pneumonia, hypoxemia and atelectasis) within the first seven hospital days, which was defined as development of one or more of the following: [1] pneumonia, which was indicated by the presence of new radiographic chest infiltration and two criteria from the following list: dyspnea, cough with purulent sputum, temperature > 38 °C, altered respiratory auscultation, leukocytosis > 14,000/ml or leucopenia < 3000/ml; [2] hypoxemia, which was defined as peripheral oxygen saturation (SpO_2_) ≤ 90%, with a requirement for administering or elevating supplemental oxygen to keep SpO_2_ ≥ 90%; and [3] evidence of atelectasis from radiological evaluation, in association with dyspnea. (B) The number of days of the hospital stay, until discharge based on the physician's decision, was also assessed. The patients and the assessor of the outcome measurements were blinded to the group allocation.

### Intervention

The treatment program was administered at a frequency of two sessions daily (in the morning and late afternoon) until the seventh postoperative day, starting from the first day. Early mobilization started from the first day.

AD treatment consists of maximum expiratory airflow with tidal breathing at various lung volumes, to mobilize expectoration while decreasing coughing episodes.^15^ During the treatment session, the subjects were in a semi-reclining position (at a 45° angle). The treatment sessions consisted of three stages: (1) unsticking the secretions at low volume; (2) collection at medium volume; and (3) evacuation by breathing at high volume. When sufficient mucus had reached the upper airways, the mucus could then be expectorated by means of a cough or a huff.^16,17^

First, the physiotherapist placed his hands over the subject's chest to follow the breathing pattern and avoid paradoxical breathing. In the first stage, the subject started with diaphragmatic breathing at low lung volume with the following cycle: (a) inspiration slowly through the nose; (b) pause of three seconds between inspirations and expirations to permit the air to get behind the secretion; and (c) non-forced expiration through nose or mouth in a sighing manner. This cycle was then repeated at the same lung volume until secretions were felt or heard. In the second and third stage, this cycle was repeated at medium and high lung volumes. In the end, when the mucus was felt in the larger central airways, the subjects did 2-3 effective huffs or coughs with splinting of the incision site.^15^ The cycles were then repeated until reaching a total session duration of 20 minutes.

The routine chest physiotherapy included deep diaphragmatic breathing exercises, localized breathing exercises and splinted coughing. One series of 10 repetitions of each exercise was performed with an inspiratory hold for three seconds and then relaxed expiration. The total session duration was 15 minutes.

### Data analysis

Based on the primary outcome measurements, the sample size was calculated taking into account a minimum mean difference in oxygen saturation of about 1.8% and a standard deviation of 2, as used in a previous published study,^18^ with a power of analysis of 80% and an alpha significance level of 0.05. From this calculation, it was determined that 20 participants would be required for each group. In order to allow for dropouts, a total of 60 subjects were recruited.

Continuous variables that were normally distributed were presented as the mean ± standard deviation (SD). Paired and unpaired-sample t tests were used for comparisons within and between the groups. The Kolmogorov-Smirnov test was used to test for normal distribution of the data. An independent chi-square test was used for comparisons of categorical variables. P-values less than 0.05 were deemed to be significant.

## RESULTS

**Figure 1:**
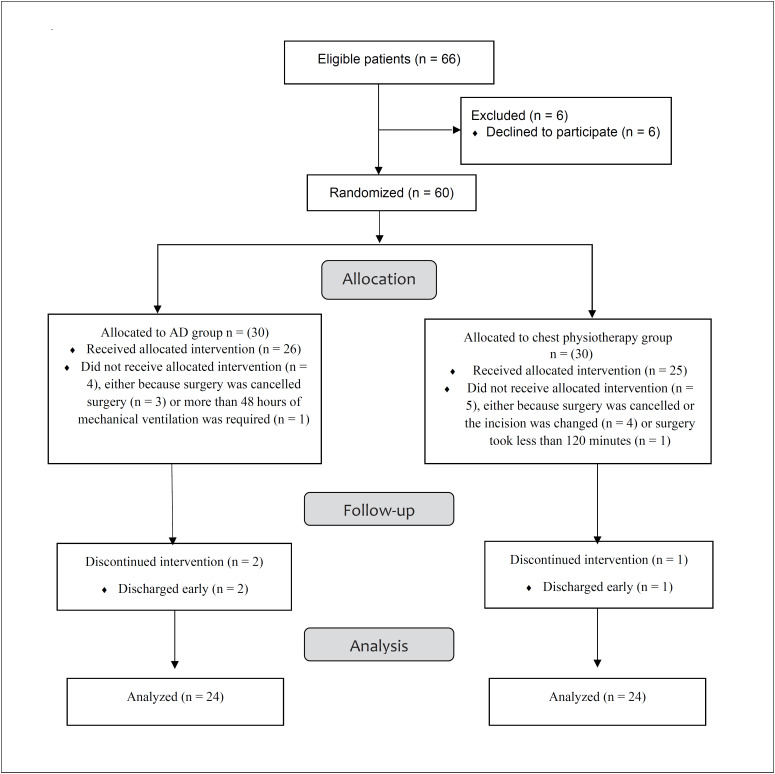
Flow of subjects through the study.

The flow of participants from recruitment to follow-up is shown in Figure 1. A total of 66 subjects were recruited and assessed for eligibility. Among these, 60 subjects were randomized equally between the two groups. Subsequently, a further six subjects were withdrawn from each group for a variety of reasons. The baseline data were comparable between the two groups, with no significant differences between the two groups in terms of demographic characteristics and clinical parameters at the pre-intervention assessment (P > 0.05), as demonstrated in Table 1.

**Table 1:** Baseline demographic and clinical characteristics of the study population. Values are in numbers (percentages) unless stated otherwise

Characteristics			AD group	Physiotherapy group
**Subjects, n = 48**				
	Age, mean ± SD, years		53.79 ± 2.395	53.88 ± 2.173
	Males, n (%)		15 (62.5)	14 (58.3)
	Body mass index, mean ± SD, kg/m^2^		32.9125 ± 2.12888	32.7240 ± 1.78535
**Clinical aspects**				
	**Smoking history, n (%)**			
		Non-smoker	6 (25.0)	9 (37.5)
		Current smoker	13 (54.2)	11 (45.8)
		Ex-smoke	5 (20.8)	4 (16.7)
	**Subjects with comorbidities, n (%)**	
		Hypertension (n)	10 (41.7)	9 (37.5)
		Diabetes (n)	11 (45.8)	13 (54.2)
		COPD (n)	3 (12.5)	2 (8.3)
	**Surgical procedure, n (%)**			
		Cholecystectomy	6 (25)	7 (29.16)
		Hernia repair	3 (12.5)	5 (20.38)
		Colectomy	2 (8.33)	3 (12.5)
		Gastrectomy	3 (12.5)	2 (8.33)
		Hepatectomy	7 (29.16)	5 (20.38)
		Pancreatectomy	3 (12.5)	2 (8.33)
		Surgical duration, mean ± SD, min	240.83 ± 15.012	238.33 ± 9.52
		Pain level, median (IQR)	4 (2-7)	4 (2-6)

AD = autogenic drainage; SD = standard deviation; COPD = chronic obstructive pulmonary disease; IQR = interquartile range.

Regarding the arterial blood gas parameters, the between-group analysis revealed that there were significant differences in post-treatment values for SaO_2_ and PaO_2_ (P = 0.0001 and P = 0.03, respectively), while no significant differences were observed with regard to HCO_3_, PaCO_2,_ and PH (P > 0.05). The within-group analysis revealed that there were significant improvements in SaO_2_, PaO_2_, PaCO_2_ and HCO_3_, with percentages of 3.12%, 9.05%, 10.1% and 10.6%, respectively, in the AD group. In the physiotherapy group, significant improvements were observed only for SaO_2_ and PaO_2_, with percentages of 1.46 % and 5.14 %, respectively. These results are demonstrated in Tables 2 and 3.

**Table 2:** Comparison of arterial blood gases parameters pre- and post-treatment in the two groups

Arterial blood gas parameters	AD group		MD	P- value	Physiotherapy group		MD	P- value
	Pre	Post			Pre	Post		
SaO_2_ (%)	94.67 ± 1.27	97.62 ± 0.87	−2.958	0.0001	93.92 ± 1.86	95.29 ± 1.33	−1.375	0.0001
PaO_2_ (mmHg)	80.08 ± 5.48	87.33 ± 4.33	−7.250	0.0001	80.17 ± 5.87	84.29 ± 5.07	−4.125	0.0001
PaCO_2_ (mmHg)	42.55 ± 6.25	38.25 ± 5.87	4.3	0.0001	41.63 ± 7.3	40.27 ± 3.03	1.362	0.348
pH	7.40 ± .056	7.39 ± 0.043	.00458	0.764	7.39 ± .08	7.43 ± .072	−0.037	0.21
HCO_3_ (meq/l)	25.5 ± 4.818	28.2 ± 4.3	−2.695	0.001	25.04 ± 2.92	26.6 ± 3.75	−1.562	0.083

MD = mean difference; AD = autogenic drainage; SaO_2_ = arterial oxygen saturation; PaO_2_ = partial pressure of oxygen; PaCO_2_ = partial pressure of carbon dioxide; HCO_3_ = bicarbonate. Data are presented as means ± standard deviations; P < 0.05 was considered to be significant.

**Table 3:** Comparison of post-treatment arterial blood parameters results between the two groups

Arterial blood gases parameters	AD group	Physiotherapy group	P-value
SaO_2_ (%)	97.62 ± 0.87	95.29 ± 1.33	0.0001
PaO_2_ (mmHg)	87.33 ± 4.33	84.29 ± 5.07	0.031
PaCO_2_ (mmHg)	38.25 ± 5.87	40.27 ± 3.03	1.41
pH	7.39 ± 0.043	7.43 ± 0.072	0.086
HCO_3_ (meq/l)	28.2 ± 4.3	26.6 ± 3.75	0.178

AD = autogenic drainage; SaO_2_ = arterial oxygen saturation; PaO_2_ = partial pressure of oxygen; PaCO_2_ = partial pressure of carbon dioxide; HCO_3_ = bicarbonate. Data are presented as means ± standard deviations; P < 0.05 was considered to be significant.

As shown in Table 4, no significant difference in the incidence of PPCs between the groups was observed (P > 0.05), but lower frequency of PPCs in the AD group was observed. There was a significant difference between groups regarding the length of hospital stay, with a shorter hospital stay among the individuals receiving AD (P = 0.0001).

**Table 4:** Incidence of postoperative pulmonary complications (PPCs) compared with length of hospital stay

Variable		AD group	Physiotherapy group	Difference between means or proportions (95% CI)	P- value
**Mean length of hospital stay ± standard deviation (days)**		7.33 ± 1.167	9.62 ± 1.610	−2.292 (-3.109 to -1.475)	0.0001
**PPCs, n (%)**		3 (12.5)	5 (20.8)	0.0833 (-0.135 to 0.296)	0.439
	Atelectasis (n)	0	0		
	Hypoxemia (n)	1	3		
	Pneumonia (n)	2	2		

P < 0.05 was considered to be significant.

## DISCUSSION

The aims of chest physiotherapy include improvement of ventilation and clearance of secretions from the airways.^19^ Evaluation of different methods for chest physiotherapy is important in order to enable the possibility of selecting the most suitable method for every patient. It becomes necessary to standardize the practical approaches, in order to assist teams in making the most suitable decision, so as to favor the subjects’ clinical outcomes. In the present study, we aimed to determine the effect of adding the AD technique to routine chest physiotherapy, in terms of improvement of gas exchange and reduction or prevention of pulmonary complications, in comparison with routine chest physiotherapy alone, among high-risk subjects undergoing UAS.

Several trials have assessed different airway clearance techniques as part of the physiotherapeutic management of ineffective cough in the postoperative period after abdominal surgery.^11,20,21^ However, only limited studies using the AD technique in the postoperative period are available.

In the present study, both treatment groups revealed significant improvements in SaO_2_ and PaO_2_ but with higher percentages in the AD group. On the other hand, no significant difference in the incidence of PPCs was observed between the two groups, although the AD group had lower frequency of pulmonary complications, and the length of hospital stay was significantly lower in the AD group than in the routine chest physiotherapy group.

Piskin et al.^22^ stated that chest physiotherapy increased oxygen saturation and returned the arterial blood gas values to normal limits. Chest physiotherapy is the main and most important treatment strategies for prevention of PPCs.^23^

The results from the present study are consistent with those of Duymaz et al.,^24^ who demonstrated that, in subjects undergoing bariatric surgery, postoperative chest physiotherapy regulated their arterial blood gases, improved oxygen saturation and respiratory functions and reduced dyspnea levels. It improved SaO_2_, PaO_2_ and pH by 15%, 18% and 7% respectively, compared with the preoperative values.

Moreover, Manzano et al.^18^ showed, in an evaluation on immediate postoperative chest physiotherapy after UAS, that there was an effective improvement in oxygen-hemoglobin saturation without any increase in abdominal pain. Furthermore, in a study evaluating chest physiotherapy and mobilization versus mobilization alone, which were applied among 74 subjects who underwent bariatric surgery, chest physiotherapy improved postoperative respiratory functions, decreased dyspnea levels, increased oxygen saturation, regulated arterial blood gases and improved functional capacity and quality of life.^24^ Additionally, Lunardi et al.^25^ compared the effect of chest physical therapy versus no treatment on the incidence of PPCs in patients who underwent esophagostomy surgery. They found that chest physiotherapy reduced the incidence of PPCs (15% versus 37%; P < 0.05), shortened the duration of antibiotic treatment and thoracic drainage and reduced the frequency of re-intubation. Also, Rocha et al. found that conventional chest physiotherapy after bariatric surgery led to improvement of tidal volume and decreased the frequency of atelectasis.^20^

To the best of our knowledge, only limited clinical trials involving use of AD postoperatively are available in the literature. The results from the present study are supported by those of a study by Shingavi et al.,^26^ who compared the active cycles of a breathing technique versus the AD technique after abdominal surgery. They found that both techniques improved chest expansion and peak expiratory flow rate, but that the active cycle of the breathing technique was more effective. Moreover, Spapen et al.^27^ studied the effect of intrapulmonary percussive ventilation physiotherapy plus assisted AD among critically ill patients on mechanical ventilation. Their patients received either intrapulmonary percussive ventilation physiotherapy plus assisted AD or conventional physiotherapy (chest wall vibrations, positioning, rib-springing, suction and aerosol therapy) or no physiotherapy. Intrapulmonary percussive ventilation plus AD tended to lower the incidence of Gram-negative infection associated with the ventilator, in comparison with the other groups.

A prospective cohort study evaluating 101 subjects postoperatively after UAS concluded that these individuals presented impaired cough effectiveness. On the first postoperative day, their peak cough flow was only 54% of the preoperative value. On the fifth postoperative day, their peak cough flow remained significantly decreased from the preoperative value (72% of that value).^3^ Furthermore, excessive sputum may be present postoperatively, due to the action of anesthetic drugs and narcotic drugs, which reduces mucociliary clearing action, diminishes lung function, increases retained secretions and leads to hypoventilation, which consequently increases the respiratory effort.^28^

In addition, abdominal surgery and obesity cause restrictive breathing patterns.^20^ Reductions in lung volume cause secretion accumulations in the airways, which are considered to be a risk factor for developing pneumonia and atelectasis. Reductions in vital capacity can also lead to atelectasis, with reduction of the partial pressure of oxygen and facilitation of alveolar collapse.^29^

Thus, there is a need for management of ineffective cough and administration of airway clearance therapy, which may possibly be provided through the improvement in blood gases and the lower incidence of postoperative complication in the AD group of the present study. AD as an airway clearance technique may be helpful in collateral airway opening through breathing with different volumes and instantaneous holding back, which leads to significant improvement in airway clearance, dyspnea, peak expiratory flow rate and mucus clearance.^30^ Airway clearance treatment generally improves gas exchange, reduces the work of breathing and clears airway secretions.^31^ Hence, removal of infected secretions in the airways can increase the ventilatory capacity and decrease direct inflammation of the airway epithelia.^32^ Lastly, autogenic drainage as an airway clearance technique could be taught to patients undergoing UAS and could be self-administered to improve these patients’ outcomes.

### Limitations

The limitations of this study were the absence of a control group that did not receive any intervention and the fact that the length of time that the subjects stayed out of bed was not assessed. Another limitation was that the physiotherapist was not blind to the study groups.

## CONCLUSION

From the results of this study, it can be concluded that adding the AD technique to routine chest physiotherapy after UAS provides improvement of blood gases, shortens the hospital stay and is accompanied by a lower percentage of pulmonary complications. Moreover, it is well tolerated by patients.

## References

[1] Weiser TG, Haynes AB, Molina G (2016). Size and distribution of the global volume of surgery in 2012. Bull World Health Organ.

[2] Weiser TG, Regenbogen SE, Thompson KD (2008). An estimation of the global volume of surgery: a modelling strategy based on available data. Lancet.

[3] Colucci DB, Fiore JF, Paisani DM (2015). Cough impairment and risk of postoperative pulmonary complications after open upper abdominal surgery. Research Support, Non-U.S. Gov’t. Respir Care.

[4] Serpa A, Hemmes SN, Barbas CS (2014). Incidence of mortality and morbidity related to postoperative lung injury in patients who have undergone abdominal or thoracic surgery: a systematic review and meta-analysis. Lancet Resp Med.

[5] Warner DO (2000). Preventing postoperative pulmonary complications: the role of the anesthesiologist. Anesthesiology.

[6] Jaber S, Chanques G, Jung B (2010). Postoperative noninvasive ventilation. Anesthesiology.

[7] Brooks-Brunn JA (1997). Predictors of postoperative pulmonary complications following abdominal surgery. Chest.

[8] Lunardi AC, Paisani DM, Silva CCBMD (2015). Comparison of lung expansion techniques on thoracoabdominal mechanics and incidence of pulmonary complications after upper abdominal surgery: a randomized and controlled trial. Chest.

[9] Smith MCL, Ellis ER (2009). Is retained mucus a risk factor for the development of postoperative atelectasis and pneumonia? -- Implications for the physiotherapist. Physiotherapy Theory and Practice.

[10] Lagerkvist AL, Sten GM, Redfors SB, Lindblad AG, Hjalmarson O (2006). Immediate changes in blood-gas tensions during chest physiotherapy with positive expiratory pressure and oscillating positive expiratory pressure in patients with cystic fibrosis. Respir Care.

[11] Allam NM, Khalaf MM, Thabet WN, Ibrahim ZM (2016). Effect of Combination of Acapella Device and Breathing Exercises on Treatment of Pulmonary Complications After Upper Abdominal Surgeries. Journal of Surgery.

[12] Lee AL, Burge AT, Holland AE (2015). Airway clearance techniques for bronchiectasis. Cochrane Database Syst Rev.

[13] McCormack P, Burnham P, Southern KW (2017). Autogenic drainage for airway clearance in cystic fibrosis. Cochrane Database Syst Rev.

[14] McCool FD, Rosen MJ (2006). Nonpharmacologic airway clearance therapies: ACCP evidence-based clinical practice guidelines. Chest.

[15] Pryor JA (1999). Physiotherapy for airway clearance in adults. European Respir J.

[16] Dab I, Alexander F (1979). The mechanism of autogenic drainage studied with flow volume curves. Monogr Paediatr.

[17] Agostini P, Knowles N (2007). Autogenic drainage: the technique, physiological basis and evidence. Physiotherapy.

[18] Manzano RM, Carvalho CR, Saraiva-Romanholo BM, Vieiro JE (2008). Chest physiotherapy during immediate postoperative period among patients undergoing upper abdominal surgery: randomized clinical trial. Sao Paulo Med J.

[19] van Kaam AH, Lachmann RA, Herting E (2004). Reducing atelectasis attenuates bacterial growth and translocation in experimental pneumonia. Am J Respir Crit Care Med.

[20] Rocha M, Souza S, Costa CMD (2018). Airway Positive Pressure Vs. Exercises with Inspiratory Loading Focused on Pulmonary and Respiratory Muscular Functions in the Postoperative Period of Bariatric Surgery. Arq Bras Cir Dig.

[21] Pasquina P, Tramèr MR, Granier JM, Walder B (2006). Respiratory physiotherapy to prevent pulmonary complications after abdominal surgery: a systematic review. Chest.

[22] Piskin O, Altinsoy B, Cimencan M (2017). The effect of bariatric anaesthesia on postoperative pulmonary functions. J Pak Med Assoc.

[23] Franco AM, Torres FC, Simon IS, Morales D, Rodrigues AJ (2011). Assessment of noninvasive ventilation with two levels of positive airway pressure in patients after cardiac surgery. Rev Bras Cir Cardiovasc.

[24] Duymaz T, Karabay O, Ural IH (2020). The Effect of Chest Physiotherapy After Bariatric Surgery on Pulmonary Functions, Functional Capacity, and Quality of Life. Obes Surg.

[25] Lunardi AC, Cecconello I, Carvalho CR (2011). Postoperative chest physical therapy prevents respiratory complications in patients undergoing esophagectomy. Rev Bras Fisioter.

[26] Shingavi SS, Kazi A, Gunjal SB, Lamuvel MW (2017). Effects of active cycle of breathing technique and autogenic drainage in patient with abdominal surgery. International Journal of Applied Research.

[27] Spapen H, Borremans M, Diltoer M (2016). Intrapulmonary percussion with autogenic drainage and ventilator-associated Gram-negative infection: A pilot study. Neth J Crit Care.

[28] Inoue J, Ono R, Makiura D (2013). Prevention of postoperative pulmonary complications through intensive preoperative respiratory rehabilitation in patients with esophageal cancer. Dis Esophagus.

[29] Sasaki N, Meyer MJ, Eikermann M (2013). Postoperative respiratory muscle dysfunction pathophysiology and preventive strategies. Anesthesiology.

[30] Lewis LK, Williams MT, Olds TS (2012). The active cycle of breathing technique: a systematic review and meta-analysis. Respir Med.

[31] Gajdos V, Katsahian S, Beydon N (2010). Effectiveness of chest physiotherapy in infants hospitalized with acute bronchiolitis: a multicenter, randomized, controlled trial. PLoS Med.

[32] Grams ST, Ono LM, Noronha MA, Schivinski CI, Paulin E (2012). Breathing exercises in upper abdominal surgery: a systematic review and meta-analysis. Rev Bras Fisioter.

